# Expressing the human proteome for affinity proteomics: optimising expression of soluble protein domains and *in vivo* biotinylation

**DOI:** 10.1016/j.nbt.2011.10.007

**Published:** 2012-06-15

**Authors:** Tracy Keates, Christopher D.O. Cooper, Pavel Savitsky, Charles K. Allerston, Claire Phillips, Martin Hammarström, Neha Daga, Georgina Berridge, Pravin Mahajan, Nicola A. Burgess-Brown, Susanne Müller, Susanne Gräslund, Opher Gileadi

**Affiliations:** 1 The Structural Genomics Consortium, University of Oxford, ORCRB, Roosevelt Drive, Oxford OX3 7DQ, United Kingdom; 2 The Structural Genomics Consortium, Department of Biochemistry and Biophysics, Karolinska Institutet, Stockholm 171 77, Sweden

## Abstract

The generation of affinity reagents to large numbers of human proteins depends on the ability to express the target proteins as high-quality antigens. The Structural Genomics Consortium (SGC) focuses on the production and structure determination of human proteins. In a 7-year period, the SGC has deposited crystal structures of >800 human protein domains, and has additionally expressed and purified a similar number of protein domains that have not yet been crystallised. The targets include a diversity of protein domains, with an attempt to provide high coverage of protein families. The family approach provides an excellent basis for characterising the selectivity of affinity reagents. We present a summary of the approaches used to generate purified human proteins or protein domains, a test case demonstrating the ability to rapidly generate new proteins, and an optimisation study on the modification of >70 proteins by biotinylation *in vivo*. These results provide a unique synergy between large-scale structural projects and the recent efforts to produce a wide coverage of affinity reagents to the human proteome.

## Introduction

Antibodies and other affinity reagents are an invaluable resource in investigating the function and distribution of proteins in addition to potential therapeutic use. Considerable efforts are being made to expand the spectrum of human proteins for which validated and selective antibodies are available. Ideally a variety of antibodies to a specific target protein should include molecules suitable for different uses, including detection in ELISA and Western blots, immunofluorescent imaging, sandwich assays, immunoprecipitation and co-crystallisation, as well as modulating the activity of target molecules in a biological context. The provision of high-quality antigens is crucial to this purpose. While short peptides may be best for eliciting antibodies to specific post-translation modifications, a better variety of antibodies are likely to be generated to larger protein fragments. The use of recombinant Protein Epitope Signature Tags (PrESTs), which are informatically derived fragments (50–150 amino acids) of human proteins, has allowed the construction of vast antigen and antibody libraries [1,2]. It has been argued that well-folded protein domains can serve as better antigens for some purposes, but this point has not been systematically explored. The provision of a large variety of such folded domains in the context of systematic affinity-reagent generating projects may shed light on these issues.

Production and characterisation of stable domains from a wide variety of proteins have been at the core of large-scale structural genomics projects. Consequently, two by-products of these projects are large collections of recombinant human protein domains (mostly preserved as expression clones and detailed protocols for expression and purification), as well as a set of methodologies for dissecting and producing new protein domains.

This report presents three aspects of the production of human protein domains. First, the existing bank of purified proteins and a summary of the methods used to obtain these proteins. Second, a description of a pilot study aimed at producing soluble domains of a set of proteins selected without regard to feasibility or prior knowledge. Finally, we present an extensive study on *in vivo* protein biotinylation, an important step in preparing proteins for immobilisation in procedures such as panning and Surface Plasmon Resonance (SPR).

## Materials and methods

### Plasmids and strains

**Figure 1:**
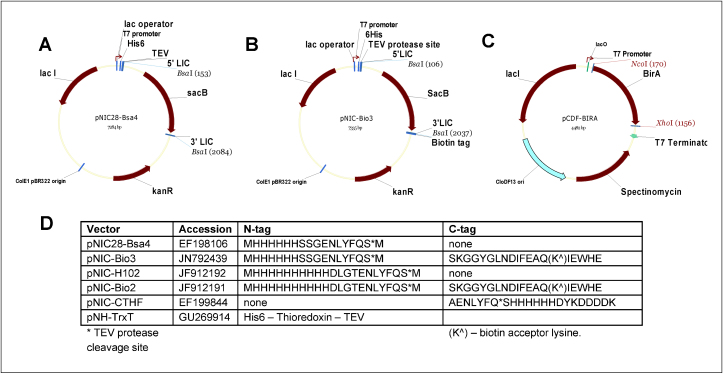
Plasmids used in biotinylation experiments. **(a)** pNIC28-Bsa4. **(b)** pNIC-Bio3. **(c)** The accessory plasmid, pCDF-BirA. **(d)** Plasmid information: GenBank accession IDs, sequence of N- and C-terminal tags. *The cleavage site for TEV protease. (K^) indicates the lysine residue that is modified with biotin.

pNIC-Bio3 and pNIC-Bio2 are kanamycin-resistance vectors that express fusion proteins with N-terminal histidine tags (His_6_ and His_10_, respectively) followed by a TEV protease cleavage site, and a C-terminal biotin acceptor site. pNIC28-Bsa4 and pNIC-H102 are identical to pNIC-Bio3 and pNIC-Bio2 respectively, but lack the C-terminal tag. All vectors are suitable for ligation-independent cloning as described [3]; more vector details are provided in Fig. 1.

The *Escherichia coli* BirA gene, encoding biotin-protein ligase, was cloned into plasmid pCDF-DUET1 (Novagen; spectinomycin-resistance), creating the plasmid pCDF-BirA.

The expression host strain BL21(DE3)-R3-pRARE2 is a phage T1-resistant strain bearing a plasmid (pRARE2; chloramphenicol-resistance) that provides rare-codon tRNAs [3]. This strain was transformed with pCDF-LIC and colonies were selected on media containing chloramphenicol (34 μg/ml) and spectinomycin (50 μg/ml) to create the strain Rosetta-R3-BirA, which was used as host in biotinylation experiments.

The plasmid sequences have been deposited with the following accession numbers: pNIC-Bio2 (GenBank ID: JF912191), pNIC-Bio3 (GenBank ID: JN792439), pNIC-H102 (GenBank ID: JF912192), pNIC28-Bsa4 (GenBank ID: EF198106) and pCDF-BirA (GenBank ID: JF914075).

### Overview of protein production methods

The methods used for cloning, protein expression and purification are summarised briefly here; full details have been published (intracellular proteins [3,4]; secreted proteins in bacteria [5] and baculovirus [6]).

Multiple constructs of every target gene were cloned in parallel as PCR fragments, using ligation-independent cloning (LIC). The cloning vectors for *E. coli* included fusion tags for affinity purification, typically N-terminal His_6_ tags that can be cleaved with Tobacco Etch Virus (TEV) protease. After clone verification, the plasmids were used to transform an expression strain, typically a derivative of Rosetta2 (a BL21 derivative harbouring the plasmid pRARE2 that provides 7 rare-codon tRNAs; Novagen). All clones were tested in small-scale cultures in rich medium (TB or LB), and protein expression was induced by IPTG or arabinose at low temperatures (15–25°C). The recombinant proteins were then purified from clarified lysates by immobilised metal affinity chromatography (IMAC) in batch, and the eluted proteins were detected by SDS-PAGE and Coomassie blue staining. Selected clones were grown and induced to a larger scale (0.75–6 L) and the proteins were purified by protocols including IMAC, gel filtration and for some proteins tag cleavage and additional steps as indicated. Proteins were analyzed by SDS-PAGE, mass spectrometry and other biophysical or biochemical means as indicated. Variations of this basic procedure include the use of different fusion tags such as C-terminal His_6_ tag, N-terminal His_6_-thioredoxin tags [3], or biotin acceptor peptides (this study) and the use of bacterial secretion vectors inducible with arabinose, with proteins purified from the culture medium [5].

### Small-scale expression tests of biotinylated proteins

Rapid, high-throughput tests for production of soluble recombinant proteins were performed using 1-ml bacterial cultures in 96 deep-well plates by a modification of an earlier method [3]. Cells were grown at 37°C in TB containing kanamycin and spectinomycin as described. When the culture turbidity reached 1–3, the temperature was reduced to 18°C. After 30 min, protein expression was induced by adding IPTG (0.1 mM) and biotin (50 or 100 μM, as indicated). Following overnight incubation, the cultures were centrifuged and the supernatant was discarded. The cell pellets could be stored frozen at −80°C or processed directly. The pellets were thoroughly suspended in 250 μl of lysis buffer comprising 100 mM HEPES, pH 8.0, 500 mM NaCl, 10% glycerol, 10 mM imidazole, 1 mg/ml lysozyme, 0.1% n-dodecyl β-d-maltoside (DDM), 1 mM MgSO_4_, 0.5 mM Tris(2-carboxyethyl)phosphine (TCEP), Benzonase (Merck; 0.5 unit/μl) and protease inhibitors (Calbiochem cocktail VI, 1:1000 dilution). The blocks were placed at −80°C for at least 20 min, then thawed in a water bath at room temperature for 10–15 min. The suspensions were mixed in a shaker at 700 rpm to effect complete lysis. The blocks were centrifuged at 3500 × *g* for 10 min. Meanwhile, Ni-NTA agarose was aliquoted (50 μl of a 50% suspension in lysis buffer) into wells of a 96-well filter plate (1.2 μm, Millipore). The clarified supernatants were transferred into the wells of the filter plate; the plate was sealed at the top and mixed for 30 min on a shaker at 400 rpm, 18°C. The liquid was then removed by vacuum filtration, taking care not to dry the beads. The beads were washed three times by adding 250 μl of wash buffer (20 mM HEPES, 500 mM NaCl, 25 mM imidazole, 10% glycerol and 0.5 mM TCEP) and vacuum filtration.

The filter plate was placed on top of a waste block (96 deep-well block) and centrifuged for 2 min at 300 × *g* to remove the remaining wash buffer. The bound proteins were then eluted by adding 40 μl of elution buffer (20 mM HEPES, pH 7.5, 500 mM NaCl, 500 mM imidazole, 10% glycerol and 0.5 mM TCEP) and mixing for 20 min at 18°C. The filter plate was placed on top of a 96-well microtiter plate and the eluates were collected by centrifugation (300 × *g*, 3 min). The eluted proteins were analyzed by SDS-PAGE and mass spectrometry as described previously [3,7].

### Expression and purification of biotin-tagged SH2 domains

Large-scale expression was performed in a custom-made expression system (LEX) (Harbinger Biotech). In this system, *E. coli* cells are cultivated in 1.5 L of medium in common 2 L glass bottles. Filtered air is bubbled through the medium at a typical rate of 4–6 L/min and thus the cultivations are both aerated and stirred. The temperature is regulated by a thermostat-controlled water bath. Inoculation cultures (20 ml) were started from glycerol stocks in TB in 100 ml Erlenmeyer flasks supplemented with kanamycin (100 μg/ml) and chloramphenicol (34 μg/ml). The cultures were incubated overnight at 30°C with shaking at 175 rpm. The following morning, bottles with 1.5 L of TB supplemented with kanamycin (50 μg/ml) and 500 μL Antifoam 204 (anti-foam agent, Sigma) were inoculated with the starter cultures. The cultures were incubated at 37°C until OD_600_ reached 2. The temperature was then reduced to 18°C and protein production was induced by the addition of 0.5 mM IPTG and 50–100 μM biotin. Protein expression was continued for approximately 20 h. Cells were harvested by centrifugation at 4500 × *g* for 10 min, resuspended in approximately 50 ml binding buffer (50 mM Na-phosphate, 500 mM NaCl, 10% glycerol, 10 mM imidazole, 0.5 mM TCEP, pH 7.5) supplemented with protease inhibitors, (Complete EDTA-free, 1 tablet/100 ml) and then stored in a freezer at −80°C.

The resuspended cells were thawed briefly with warm water and Benzonase (2000 U) was added. The suspensions were diluted in lysis buffer to approximately 100 ml before sonication (6 min, 80% amplitude, 4 s/4 s pulsing on a Sonics VibraCell) followed by centrifugation at 49,000 × *g* for 20 min. The supernatants were filtered (0.45 μm) and applied to a two-step purification procedure, IMAC and gel filtration, on an ÄKTA Xpress system (GE Healthcare). Briefly, the lysates were loaded onto a 1 ml HiTrap Chelating HP column (GE Healthcare) loaded with Ni^2+^ ions, at 0.8 ml/min. The immobilised proteins were washed first with binding buffer until stable baselines were obtained, and then with wash buffer (50 mM Na-phosphate, 500 mM NaCl, 10% glycerol, 25 mM imidazole, 0.5 mM TCEP, pH 7.5) for 20 column volumes (CV) before elution with elution buffer (50 mM Na-phosphate, 500 mM NaCl, 10% glycerol, 500 mM imidazole, 0.5 mM TCEP, pH 7.5) for 7.5 CV. The eluted proteins were collected and stored in a loop on the system, reinjected onto a gel filtration column (HiLoad Superdex 75 or 200, GE Healthcare) and finally eluted in PBS buffer (10 mM Na-phosphate, 154 mM NaCl, 0.5 mM TCEP, pH 7.5) at 1.2 ml/min. Peaks were collected in 2 ml fractions in a deep-well plate and analyzed by SDS-PAGE (Novex NuPAGE 4-12% BisTris 17w gels, Invitrogen). Relevant fractions were pooled and protein concentration was assessed by measuring the absorbance at 280 nm on a Nanodrop ND-1000 (NanoDrop Technologies) spectrophotometer. In case peaks corresponding to different multimeric states were observed in the gel filtration step, these were pooled separately. Samples from each protein batch were analyzed by electrospray ionisation mass spectrometry (ESI-MS) according to the protocol described in [8] to check the extent of the biotinylation reaction.

## Results and discussion

### Protein production and crystallisation at the SGC

The SGC has solved and deposited structures of more than 841 distinct human protein domains [9]; a similar number of other proteins have been purified but not yet crystallised (data not shown). All proteins were produced in recombinant cells, most commonly in *E. coli* but in some cases (3–4%) in baculovirus-infected insect cells. More detailed accounts of the methods and parameters used to produce and crystallise these proteins have been published [3,10]. The structures represent a highly diverse selection of proteins with a variety of metabolic, regulatory and structural functions. A rough division of the solved targets is shown in Table 1. We have attempted to cover multiple members of protein or domain families, aiming both to provide insights on biological specificity [11–18] and to build expertise in selected areas. A consequence of the family-based approach is the availability of sets of related protein domains that can be used to test the selectivity of affinity reagents [4,19,20].

Production of soluble recombinant proteins in both *E. coli* and baculovirus-infected insect cells relied on the following process:

(1) Bioinformatic analysis of the protein sequence, to predict soluble domains and their boundaries.
(2) Parallel cloning of multiple fragments, designated by the informatics analysis, into one or more expression vectors. On average, 3–5 boundaries are tested at the ends of each protein domain, resulting in 9–15 fragments. However, the number of constructs used for each target protein may be considerably larger, when the protein includes multiple domains and when more than one expression vector is used.
(3) The recombinant proteins are expressed with an affinity tag, most commonly an N-terminal hexahistidine with a cleavage site for TEV protease.
(4) Clones producing soluble protein are identified in small (1 ml) or medium (20–50 ml) scale cultures and IMAC purification. One or more clones are scaled to 1–10 L, and the proteins are purified by a sequence of IMAC, gel filtration (GF) and, when appropriate, cleavage of the tag and re-purification by IMAC.
(5) Biophysical characterisation of the proteins, including mass spectrometry (MS), GF, and thermostability analysis.

**Figure 2:**
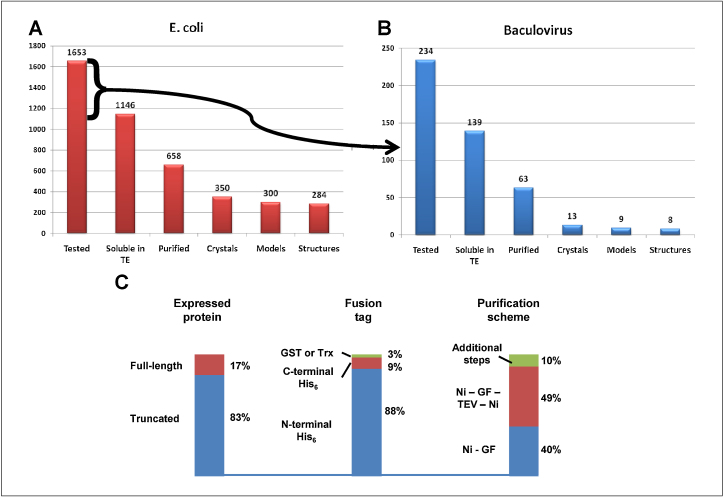
Overview of expression and purification statistics (SGC-Oxford, 2004–2009). **(a)** Pipeline of targets tested in *E. coli*. The bars represent the number of targets (proteins) that were tested; targets which showed production of soluble protein in small scale test expression; targets that were purified from large-scale culture; targets that generated diffracting crystals; initial models; and finished structures. **(b)** Targets that failed to express as soluble proteins in *E. coli* were subcloned into baculovirus vetors and expressed in insect cells. The bars denote the same data as in (a). **(c)** Summary of the characteristics of constructs and purification schemes used for the proteins that crystallised successfully. Full-length proteins include those with ‘trivial’ truncations (deletion of membrane-spanning, targeting signals or 1–2 residues from either end). The large tags (GST or Trx) were cleaved before crystallisation. Purification schemes: Ni – IMAC purification. GF – gel filtration. Ni-GF-TEV-Ni: IMAC and gel filtration, followed by cleavage of the His_6_ tag and removal of contaminating proteins be re-binding to the IMAC resin. Additional steps include a diversity of chromatographic methods.

The entire set of protein domain structures solved by the SGC, including the sequences of constructs and the full methods, are continuously updated in the web site [9]; a snapshot list (January 2011) is provided in supplementary table S1. All clones are available upon request from the SGC or from partner distributors, as described in the web site. A detailed analysis of a subset of protein domains produced at the Oxford SGC has been published [3], also providing guidelines to construct design. Figure 2 shows the success rates in expressing human protein domains in *E. coli* (Fig. 2a) and in insect cells (Fig. 2b). Figure 2c summarises the approaches used for production and purification of the proteins that yielded crystal structures. A clear outcome of the parallel testing of multiple constructs has been the identification, for a large fraction of targets, of domains that can be expressed as stable, soluble proteins in relatively high yields. Once optimal constructs are identified, purification of most protein domains can be achieved using standardised procedures. All the truncated proteins represent intact, independently folded domains; these comprise enzymatic (e.g. kinase, dehydrogenase, phosphatase), molecular recognition (e.g. PDZ, 14-3-3, SH2) or regulatory (e.g. RGS) domains [3].

### Processing newly prioritised targets

When facing new proteins that emerge from genetic studies or pathway analyses, the impact of the accumulated experience of the SGC and similar organisation can be two-fold. First, many of the new genes of interest may already have been produced in the SGC; alternatively, for novel targets, the well-tried methods can be used to rapidly generate and identify constructs that produce soluble proteins. To test this, we attempted to handle a set of non-membrane proteins suggested by collaborators, with no consideration of prior work at the SGC or of predicted tractability. The 15 selected proteins were new to us; a few were purified previously by other groups, while some were not reported to be purified. Table 2 summarises the cloning and testing processes performed on each of the targets, within a time frame of three months.

In all cases, we have applied our standard construct design and evaluation principles regardless of prior knowledge; when a protein structure was known, the designed construct boundaries closely clustered around the published structural boundaries. Constructs for cytoplasmic expression were cloned in parallel into four vector systems [3]: the *E. coli* vectors pNIC28-Bsa4 (N-terminal His_6_ tag), pNIC-CTHF (C-terminal His_6_ + Flag tag), pNH-TrxT (N-terminal His_6_/Thioredoxin tag) and the baculovirus transfer vector pFB-LIC-Bse (N-terminal His_6_ tag). Four targets encoding extracellular or secreted domains were expressed in *E. coli* as fusions to the bacterial secreted protein OsmY [5]. Equivalent constructs were cloned into a baculovirus transfer vector, fused to a signal peptide of baculovirus gp64.

Table 2 represents the results, which are typical of the parallel approach used in structural genomics. Using just a default vector (N-terminal His_6_ tag) in bacteria provided soluble constructs for the majority of cytoplasmic targets tested. One gene (RCC1) only yielded substantial levels of soluble expression in *E. coli* with either the C-terminal tag vector or a large fusion tag (thioredoxin), and another (TEX9) was only soluble with the C-terminal tag vector. Two other genes (PLEKHH1 and the enzymatic domain of HCK) could only be expressed in bacteria with a thioredoxin tag. The recently introduced OsmY fusion [5] allowed the production of secreted domains of SPON1, CST3 and FAT3; the secreted proteins could be harvested in approximately equal amounts from the culture supernatants and from the periplasm. One construct was selected from each gene for large-scale (1–4 L) purification; all proteins showed well-defined peaks on gel filtration, and were confirmed by mass spectrometry. Finally, expression of ITGBV (Integrin β5) was attempted in insect cells as a near full-length protein, in combination with integrin α5, but the levels were marginal. This target may require more extensive optimisation or expression as fragments.

In summary, we have been able to produce soluble domains of 14 out of 15 targets tested, with yields of several milligrams. This, together with earlier data from the SGC and others, demonstrates the feasibility of providing soluble domains for the majority of novel targets emerging from genetic and systematic studies of disease pathways.

### *In vivo* biotinylation

Biotinylation of the antigen is often the method of choice for protein immobilisation for selecting and evaluating affinity reagents. *In vitro* biotinylation is frequently used, whereby lysine residues in the antigen are chemically modified. However, biotinylation of a short acceptor peptide *in vivo* is an attractive method to achieve site-specific modification without the risk of interfering with protein folding or function of the antigen. *In vivo* biotinylation is achieved by co-expressing the protein of choice (fused to a biotin acceptor peptide) and the bacterial biotin-protein ligase (BirA) in the presence of biotin. To investigate factors that affect the yield and homogeneity of biotinylated proteins, we tested a set of 24 human proteins using two vector systems. Biotin acceptor tags were added at the C-termini, and oligohistidine sequences (cleavable with TEV protease) were added at the N-termini for protein purification. Based on our sporadic observations, we tested both hexahistidine (His_6_) and decahistidine (His_10_) tags. The latter could be useful for some applications (e.g. SPR), but may decrease protein yields because of aggregation.

24 diverse human protein domains (Table 3) were cloned into each of four vectors, generating combinations of His_6_ or His_10_ tags, with or without biotinylation sites (vectors pNIC-Bio3, pNIC-Bio2, pNIC28-Bsa4 and pNIC-H102, see Fig. 1). Soluble protein production was tested in triplicate small-scale cultures in the presence of 100 μM biotin; the yield of soluble protein was evaluated using SDS-PAGE of fractions eluted from Ni-NTA beads. In separate experiments, the clones in the His_6_ vectors pNIC28-Bsa4 and pNIC-Bio3 were tested in the absence of biotin or in presence of 50 and 100 μM biotin.

**Figure 3:**
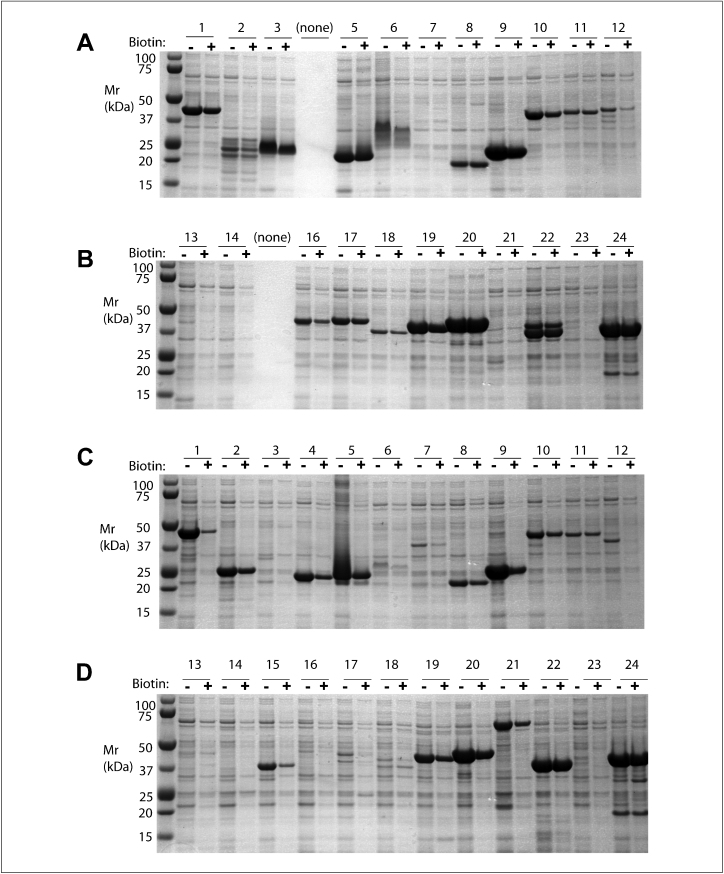
Effect of biotin in culture media on expression of biotin-tagged proteins. The 24 genes listed in Table 3 were cloned into vectors pNIC28-Bsa4 (panels A and B) and pNIC-Bio3 (panels C and D). 1-ml cultures of each clone were induced in presence (+) or absence (−) of 50 μM biotin in the culture medium. The recombinant proteins were extracted and purified by IMAC, resolved by SDS-PAGE gradient gels, and stained with Coomassie blue. The recombinant proteins can be identified according to the predicted masses listed in Table 3; some proteins show aberrant mobility, possibly due to heterogeneous modification or proteolysis. (*Note*: the lanes marked ‘none’ indicate missing clones.)

Figure 3 shows a representative experiment, comparing protein production in absence (lanes marked ‘−’) and presence (‘+’) of 50 μg/ml biotin. Panels A and B show expression of protein domains cloned in pNIC28-Bsa4, lacking a biotinylation signal. In general, the intensity of the stained bands in each pair of lanes (−/+ biotin in the growth medium) is similar. Panels C and D show expression of protein domains cloned in pNIC-Bio3, which contain a C-terminal biotin acceptor site. Here, the picture is different: A fraction of clones (e.g. clones 1, 4, 5, 9, 12, 15) show significantly lower yield (2–7-fold) of protein in the presence of biotin. Furthermore, some clones (e.g. 17, 18) yield very little protein with the C-terminal tag compared with the untagged protein, regardless of the biotin concentration in the culture medium. These effects are protein-specific, as several clones (e.g. 8, 10, 11, 22 and 24) are indifferent to the presence of biotin. The results of this (and replicate) experiment(s) are summarised in Table 3.

**Figure 4:**
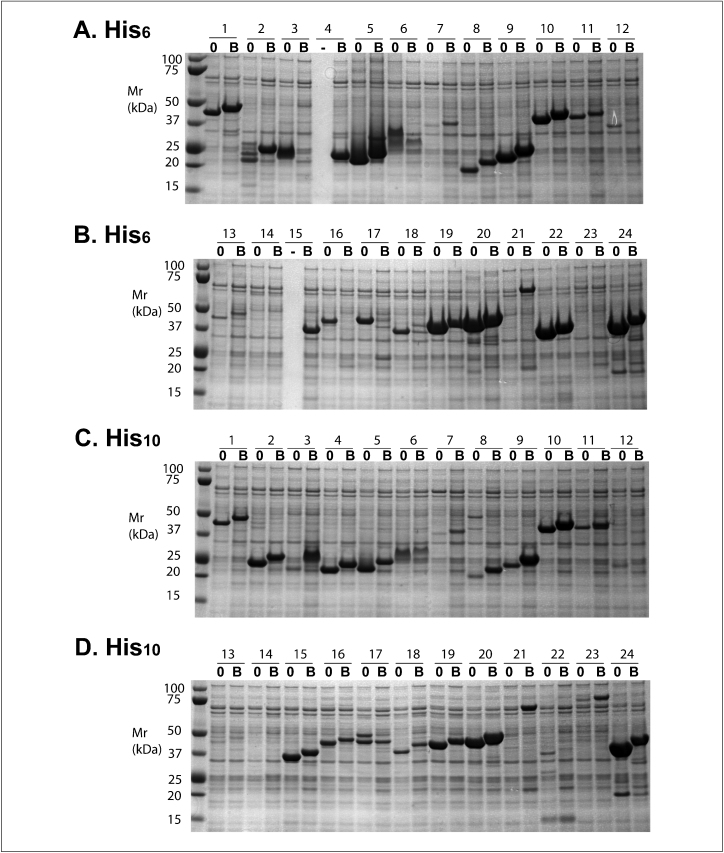
Effect of the N-terminal tag on protein yields. The 24 genes listed in Table 3 were cloned into vectors with a His_6_ tag (panels A and B) or a His_10_ tag (panels C and D). Each pair of adjacent lanes differ by the absence (0) or presence (B) of a C-terminal biotin tag, which adds 2.6 kDa to the protein mass. In panels A and B, the genes are cloned into pNIC28-Bsa4 (lanes 0) and pNIC-Bio3 (lanes B). In panels C and D, the genes are cloned into pNIC-H102 (lanes 0) and pNIC-Bio2 (lanes B). 1-ml cultures of each clone were induced in presence of 100 μM in the culture medium. The recombinant proteins were extracted and analyzed as in Fig. 3.

Figure 4 shows a representative experiment comparing the two N-terminal purification tags: His_6_ (panels A and B) and His_10_ (panels C and D). In each pair of lanes, the lane marked ‘0’ is the protein lacking the C-terminal biotin tag, and the lane marked ‘B’ is the C-terminal tagged protein. The difference in size between each pair represents the 2.6 kDa tag (peptide + biotin). Comparing the recovery of purified proteins from the His_6_ and His_10_ vectors (panels A vs. C and B vs. D), the results are gene-specific. However, there is a tendency for lower yields of the His_10_-tagged proteins relative to the His_6_-tagged counterpart.

**Figure 5:**
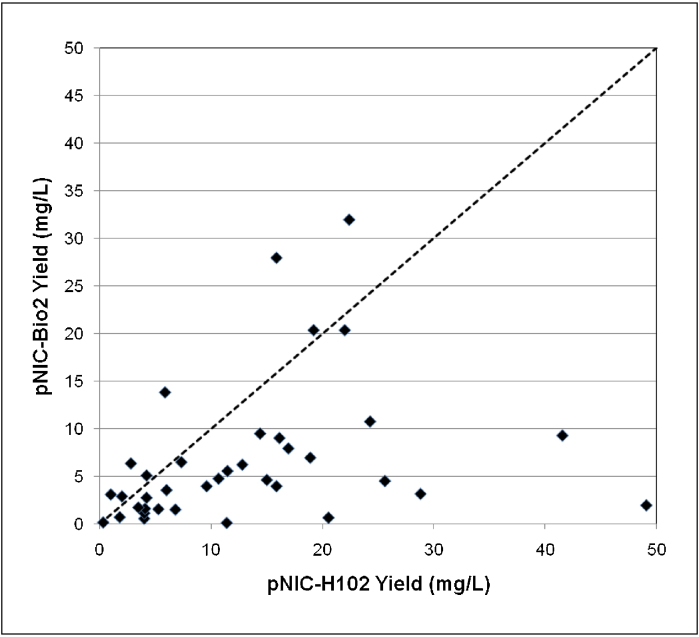
Comparison of expression of SH2 domains with (*Y*-axis) or without (*X* axis) a C-terminal biotin acceptor tag. 35 human SH2 domains were cloned into the vectors pNIC-H102 (no biotin tag) and pNIC-Bio2 (C-terminal biotin tag). Each of the resulting 70 clones was used in a 1.5-Litre expression culture, in presence of 50 μM biotin. The proteins were purified and the yields were measured as described. Each spot represents the yields from one gene in both vectors, in mg of purified protein/L of culture. The dotted line (*x* = *y*) is overlaid to indicate that, for most proteins, the yield of biotinylated protein is lower than the corresponding protein lacking the biotin tag.

Small-scale experiments provide only a semi-quantitative estimate of protein yields. We tested a separate set of 35 SH2 domains cloned into pNIC-H102 and pNIC-Bio2, at a production scale of 1.5 L (in the presence of 50 μM biotin). The proteins were purified using a standard two-step procedure (IMAC and gel filtration), and the yields were measured. Figure 5 shows the comparison of the yields of proteins expressed with or without the C-terminal tag. The graph shows the considerable scatter of the results; most of the points are below the diagonal (*x* = *y*; dotted line), illustrating that the yield of biotinylated proteins is usually lower than that of the corresponding clone lacking the biotin acceptor peptide. The average reduction in yield is only 30%, but 6/35 clones tested showed more than 5-fold reduction in yield. Although low yields can be overcome by increasing culture volumes, it may be worth testing in individual cases whether the biotin tag affects the stability or solubility of the purified protein.

**Figure 6:**
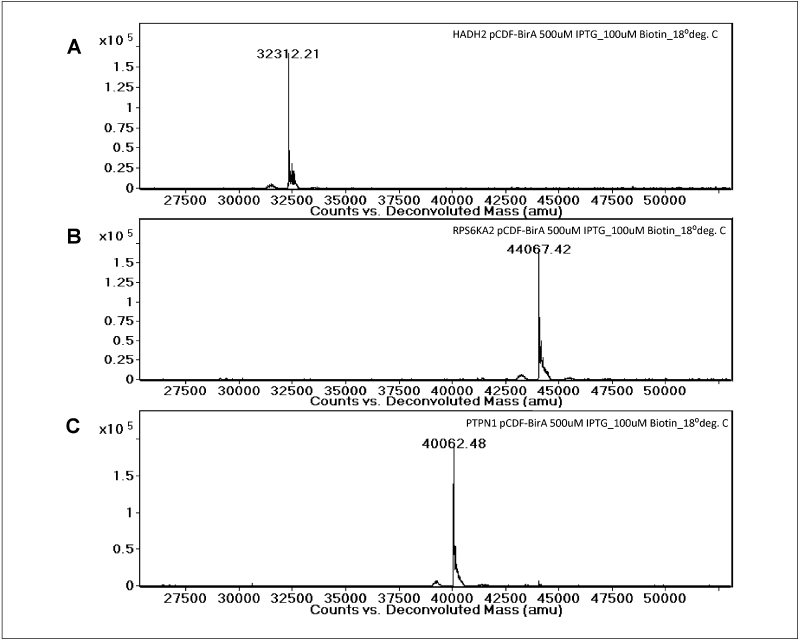
Mass spectrometric (MS) analysis of biotinylated proteins. Three proteins expressed in *E. coli* from vector pNIC-Bio2 in combination with pCDF-BirA were purified and analyzed by LC–ESI-MS. The expected masses of the three proteins (including a single biotin modification) are: **(a)** HADH2: 32,310 Da, **(b)** RPS6KA2: 44,064 Da, **(c)** PTPN1: 40,060 Da. The deconvoluted mass spectra are indicative of homogeneously modified proteins. The observed masses (32,312, 44,067 and 40,062, respectively) are as expected within 3 Da; the reasons for the small mass deviations are unclear.

The precise masses of the purified proteins were evaluated using mass spectrometry (representative results are shown in Fig. 6). For all proteins expressed with the biotin acceptor tag, >90% of the purified protein was biotinylated. We could also obtain mass measurements for the highly expressed proteins purified from the small-scale cultures. In all cases, fully biotinylated proteins were observed when the culture medium included 50 or 100 μM biotin. No biotinylation was seen when the medium did not include added biotin. The lower concentration of biotin is sufficient for full biotinylation of all proteins included in this study; however, we have encountered a small number of (highly expressed) proteins where higher concentrations of biotin were required.

The optimal procedure that emerges from these biotinylation experiments and other experiments not shown are: (1) Addition of a biotin acceptor tag can affect protein expression in unpredicted ways, often leading to reduced yields. (2) Addition of 50 μM biotin to the culture medium is generally sufficient to achieve full biotinylation, although special cases of highly expressed genes may required adding 100 μM or more. (3) A host strain that expresses BirA as well as rare-codon tRNAs gives optimal, consistent results for eukaryotic genes. (4) As always with protein production, individual proteins may require specific optimisation of the induction, extraction and purification conditions.

## Concluding remarks

Earlier studies have shown the synergy between the protein-producing capacity of structural biology and high-throughput production of affinity reagents [4,20]. In the original studies [4,20], a set of purified protein domains from the SH2 family was produced, and recombinant or monoclonal binders to most of them were obtained within a short time span. The panel of related protein domains provided an excellent platform for assessing the selectivity and the binding affinities of the binders.

The present work explores the possibility of extending the antigen space to a wider variety of human proteins, especially those associate with disease or with signalling networks. The panels of purified proteins produced through the activity of large-scale structural biology programs already include >1000 human proteins of interest. The small pilot study reported here shows that processing of new proteins to generate soluble domains can be achieved with high efficiency. Finally, the initial results of transferring previously expressed proteins to an *in vivo* biotinylation system show that, although the protein yields are sometimes lower, it is possible to routinely achieve complete biotinylation of all proteins tested.

The ability to rapidly provide purified proteins (and, subsequently, affinity reagents) from novel genes that emerge from functional and genetic studies, can provide a major opportunity to understanding the roles of these proteins and their suitability as targets for clinical intervention.

## Figures and Tables

**Table 1:** Distribution of human protein structures determined at the SGC

Target area	Number of proteins
Protein kinase	89
Oxidoreductase	87
Transferase	78
Ubiquitilation	65
Miscellaneous	55
G-protein regulator	45
GTPase	43
Linker-PDZ	37
Nucleotide metabolism	34
Phosphatase	32
Methyl-lysine reader	28
Bromodomain	25
Non-protein kinase	21
RNA and DNA helicases	15
aa metabolic enzymes	13
Hydrolase-other	13
Isomerase	13
Protease	13
Cytoskeleton	12
PARP	11
BTB-Kelch	10
Lipid signalling-other	10
GTPase-RAS	9
Metabolic-NonDR	9
Other-signal	9
PI signalling – lipid binding domains	9
ATPases – Hsp70	6
GPCR-extracellular domains	6
WD40	6
Apoptosis-inflammation domains	4
Cytochrome P450	4
Lyase-carbonic-anhydrase	4
Macro domain	4
SOCS-box-containing	4

All structures of distinct human proteins or domains were divided into biochemical areas, defined either by structural similarity or involvement in biological processes. The larger groups may encompass highly diverse proteins. A full list of structures including experimental procedures is provided on-line at www.thesgc.org/structures.

**Table 2:** Expression of soluble domains of newly introduced targets

Target gene/Uniprot ID	Soluble constructs/tested				Domains produced (aa range)	Prior status
	N-His	C-His	His/Trx	Baculo		
AMPH/P49418	5/7	5/8	4/8	8/8	BAR domain (aa34–236)	67% identity to human Bin1 [21]
CNO/Q9NUP1	2/7	0/9	5/7	3/7	(aa1–217)	None reported
ETV1/P50549	5/8	0	7/7	7/7	PEA3, ETS-N-terminal domain (aa223–326)	Short homology to ets-1
HCK (SH2-SH3)/P08631	6/8	2/8	5/8	4/7	SH3 domain (aa72–138)	Structure solved (PDB:3NHN) [22]
HMHA1/Q8IYN3	3/13	(weak)	1/12	5/13	FCH: Fes/CIP4 homology domain (aa254–521)	31% homology in RhoGAP domain
INADL/Q8NI35	3/5	1/6	2/5	4/5	PDZ 2 + 3 (aa254–530)	PDZ domain structures solved by NMR (PDB 2DB5, 2DMZ and 2DAZ)
MSX2/P35548	5/8	2/9	7/8	3/8	Homeobox domain (aa143–211)	Highly homologous to MSX1 in homeobox
RBM3/P98179	6/6	5/6	6/6	6/6	RRM1 (aa1–106)	68% identity to closest PDB homologue 1X5S
RCC1/P18754	0/1	1/1	1/1	1/1	Full-length RCC1 (aa1–421)	Structure solved (PDB:1A12) [23]
TEX9/Q8N6V9	0/3	2/4	(weak)	(weak)	(aa172–391)	None reported
PLEKHH1/Q9ULM0	0/16	0/16	4/16	ND	Refolded MyTH4 domain (aa832–990)	None reported
HCK (kinase)/P08631	0/4	ND	5/6	(weak)	SH3_SH2_Kinase (aa72–526)	Structure solved (PDB:1QCF); expressed in mammalian cells [24]

Secreted proteins				
Target gene	*E. coli*	Baculo		
SPON1/Q9HCB6	1/3	1/6	Spondin_N domain(aa194–413)	Structure solved (aa1–200), PDB:3COO [25]
CST3/P01034	1/1	1/1	Cystatin domain (aa27–146)	Structure solved (PDB: 1G96). Periplasmic expression in *E. coli*[26]
FAT3/Q8TDW7-3	11/24	ND	Cadherin 32 (aa3443–3553); EGF_like (aa4016–4137)	None
ITGB5/P18084	ND	(weak)	ND	55% identical to integrin beta-3 structure (PDB:3IJE), expressed as complex with ITGAV in insect or mammalian cells [27,28]

For each target and vector, the number of constructs expressing soluble proteins is listed, out of the number of constructs tested. ‘Domains produced’ indicates the segment of the target protein included in a construct that was selected for scale-up and purification. N-His, C-His, Trx and Bacolu refer to clones in the vectors pNIC28-Bsa4, pNIC-CTHF, pNH-TrxT and the baculovirus transfer vector pFB-LIC-Bse [4].

**Table 3:** Genes used in biotinylation experiments

Number	Gene	Gene family	Mr (kDa)	Biotin/unmodified
1	ARHGEF2	G-protein-GEF	47.2	–
2	CBPP22	EF hand	25.0	0
3	CDC42	GTPase-RHO	23.8	N/D
4	CENTG1	GTPase-RAS	22.0	–
5	DIRAS2	GTPase-RAS	22.1	–
6	DIRAS1	GTPase-RAS	24.9	–
7	DUSP16	Phosphatase-Dual-Spec	36.6	–
8	DUSP16	Phosphatase-Dual-Spec	18.7	–
9	GEM	GTPase-RAS	23.8	–
10	GNAI3	GTPase-Trimeric	42.6	0
11	MAP2K2 (MEK2)	Kinase-STE	42.3	0
12	MAP2K3 (MEK3)	Kinase-STE	37.1	–
13	MAP2K4 (JNKK1)	Kinase-STE	43.3	N/D
14	MAP2K5 (MEK5)	Kinase-STE	36.0	N/D
15	MAP2K6 (MEK6)	Kinase-STE	35.2	–
16	MAP2K7 (JNKK2)	Kinase-STE	42.9	N/D
17	MAPK3 (ERK1)	Kinase-CMGC	45.7	N/D
18	MAPK6 (ERK3)	Kinase-CMGC	40.2	N/D
19	MAPK8 (JNK1)	Kinase-CMGC	44.5	–
20	MAPK9 (JNK2)	Kinase-CMGC	46.3	–
21	PAK4	Kinase-STE	66.6	–
22	PAK7	Kinase-STE	38.0	0
23	PAK6	Kinase-STE	77.4	–
24	PPP1R12B	Phosphatase-regulatory	39.0	0

A list of the human genes used in the experiments shown in Figs. 3 and 4. The numbering corresponds to the annotations in the figures. The molecular weights are of the proteins produced from the vector pNIC28-Bsa4, which include a His_6_-tag only. The C-terminal biotinylation tag adds 2.6 kDa.

The column marked Biotin/unmodified summarises the results of test expression of the C-terminal tagged proteins, comparing the yield in presence and absence of biotin in the growth medium (Fig. 3 and similar experiments). ‘0’ indicates no effect, ‘–’ indicates a reduction in yield in the presence of biotin, and N/D indicates that the yields were too low to compare.
